# A Case of Misdiagnosed Cesarean Scar Pregnancy with a Viable Birth at 28 Weeks

**DOI:** 10.1155/2014/375685

**Published:** 2014-10-14

**Authors:** Sakiko Nukaga, Shigeru Aoki, Kentaro Kurasawa, Tsuneo Takahashi, Fumiki Hirahara

**Affiliations:** ^1^Perinatal Center for Maternity and Neonate, Yokohama City University Medical Center, 4-57 Urafunecyou, Minami-ku, Yokohama, Kanagawa 232-0024, Japan; ^2^Department of Obstetrics and Gynecology, Yokohama City University Hospital, 3-9 Fukuura, Kanazawa-ku, Yokohama, Kanagawa 236-0004, Japan

## Abstract

We report our experience with a case of presumptive cesarean scar pregnancy, based on detection of a gestational sac (GS) in early pregnancy at the site of a previous cesarean scar. The GS grew into the uterine cavity as the pregnancy progressed, showing an ultrasound image similar to that of a normal pregnancy. Thus, the pregnancy continued, resulting in a viable birth at 28 weeks of gestation. Cesarean scar pregnancy is classified as myometrial implantation or implantation growth into the uterine cavity. In the latter type, the gestational sac moves upward with increasing gestational weeks and it shows the same ultrasound image as a normal pregnancy. Therefore, the diagnosis must be made in the early pregnancy.

## 1. Introduction

A caesarean scar pregnancy (CSP) is a very rare type of ectopic pregnancy that becomes implanted in a C section scar. It has an estimated incidence of 1 : 1800–2200 pregnancies [1, 2]. Two types of cesarean scar pregnancy have been reported. The first type, a deep implantation in a cesarean scar defect towards the bladder and the abdominal cavity, is associated with a high risk of uterine rupture, uncontrollable bleeding, hysterectomy, and maternal morbidity; the second involves an implantation growing into the uterine cavity [3]. The former type of cesarean scar pregnancy, with deep myometrium implantation, is more likely to cause uterine rupture even in early pregnancy [4, 5]. In principle, it is recommended that the pregnancy be terminated. On the other hand, several reports have described the latter, with growth into the uterine cavity, as resulting in viable births if the pregnancy is allowed to continue [6–10].

We report our experience with a case of presumptive cesarean scar pregnancy, based on detection of a gestational sac (GS) in early pregnancy at the site of a previous cesarean scar. The GS grew into the uterine cavity as the pregnancy progressed, showing an ultrasound image similar to that of a normal pregnancy. Thus, the pregnancy continued, resulting in a viable birth at 28 weeks of gestation.

## 2. Case Report

**Figure 1:**
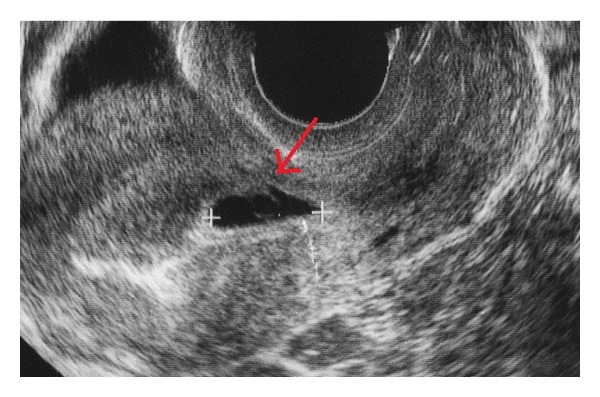
At 6 weeks and 1 day. A wedge-shaped gestational sac (GS) at the site of a previous cesarean scar.

**Figure 2:**
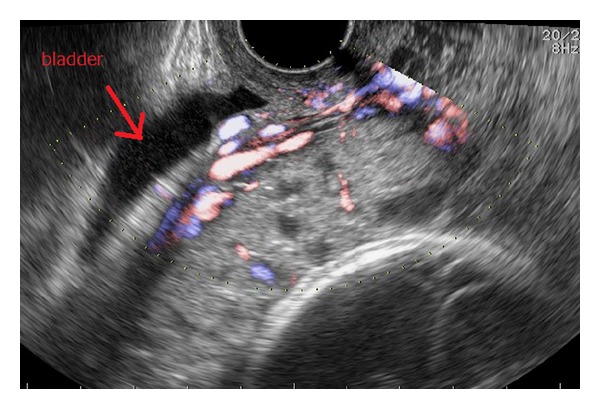
At 24 weeks of gestation. Transvaginal ultrasonography revealed loss of hypoechoic appearance of the retroplacental zone, lacunas in the placenta, and bulging of the bladder.

The patient was a 35-year-old, gravida 2, para 1, woman who had undergone cesarean delivery by low transverse incision because of cephalopelvic disproportion 4 years previously. She was referred to another hospital with suspicion of a cesarean scar pregnancy because a wedge-shaped gestational sac (GS) was found at the scar in the lower uterine segment at 6 weeks and 1 day of gestation (Figure 1). Three days later, a deformed GS at the previous uterine scar was confirmed and she was closely followed up due to the potential for miscarriage. At 9 weeks of gestation, the deformity had disappeared with growth of the GS into the uterine cavity. The ultrasound image was similar to that of a normal pregnancy and the gestation was allowed to continue. She was referred to our hospital with a diagnosis of total placenta previa at 24 weeks of gestation. Transvaginal ultrasonography revealed loss of hypoechoic appearance of the retroplacental zone, lacunas in the placenta, and bulging of the bladder (Figure 2). Magnetic resonance imaging showed disappearance of the sonolucent zone between the myometrium and the bladder, a heterogeneous signal from the internal placenta, and an irregular bulge with a flow void on the surface of the bladder on T2-weighted images. Cystoscopy revealed normal bladder mucosa. Based on these findings, placenta previa-accreta was suspected. She did not desire to preserve her uterus. Therefore, she agreed to a cesarean hysterectomy without attempting placenta removal after delivery of her baby, if placenta accreta was strongly suspected. At 28 weeks of gestation, she was hospitalized because of warning bleeding, and administration of tocolytic agents was initiated.

Five days later, preterm premature rupture of membranes developed with intense uterine contractions, necessitating an emergency cesarean section.

**Figure 3:**
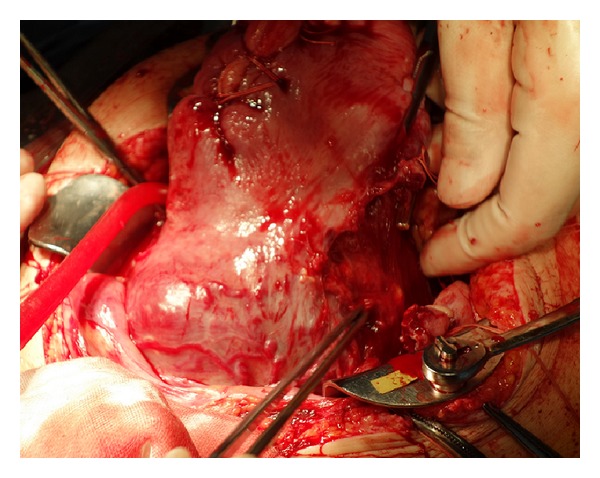
Operative findings. After delivery of the baby through a vertical incision in the uterine corpus, no myometrium was detected in the lower uterus and the placenta was visible through the uterine wall; these findings were consistent with a cesarean scar pregnancy.

With consideration of a possible cesarean hysterectomy, a ureteral stent was placed after induction of spinal anesthesia. An intra-arterial balloon catheter could not be prophylactically placed, because it was an emergency operation. Intraoperatively, no myometrium was detected in the lower uterus and the placenta was visible through the uterine wall, findings consistent with a cesarean scar pregnancy (Figure 3). Placenta percreta was diagnosed and cesarean hysterectomy was indicated. A viable baby was delivered after classical uterine incision followed by abdominal total hysterectomy without removal of the placenta. The bladder musculature strongly adhered to the incision scar of the previous cesarean section. There was massive hemorrhaging with detachment of the bladder, requiring partial resection of the bladder musculature. The bleeding volume was approximately 6.5 L and a massive blood transfusion was required. Her postoperative course was uneventful and the patient was discharged from the hospital 7 days after the operation. The pathological examination confirmed placenta percreta.

## 3. Discussion

This case highlights two points: CSP with implantation growth into the uterine cavity can be diagnosed only in the very early stage of pregnancy and it will eventually result in placenta previa-accreta.

Firstly, it was elucidated that CSP with implantation growth into the uterine cavity can be diagnosed only in the very early stage of pregnancy. As the clinical course of our patient shows, in CSP cases with implantation growth into the uterine cavity, the GS can be observed over the scar at the uterine incision very early in the pregnancy, making it possible to differentiate from a normal pregnancy. However, the GS moves upward with increasing gestational weeks, presenting the same ultrasound image as a normal pregnancy. Therefore, diagnosis in the early stage of pregnancy is very important for CSP with implantation growth into the uterine cavity, and it is necessary to explain to patients that pregnancy following a cesarean section requires a hospital visit early in the pregnancy.

Secondly, CSP with implantation growth into the uterine cavity will eventually result in placenta previa-accreta. Recent epidemiological studies have also found that the strongest risk factor for placenta praevia is a prior caesarean section suggesting that a failure of decidualization in the area of a previous uterine scar can have an impact on both implantation and placentation [11]. Although there have been a few reports of CSP with implantation growth into the uterine cavity which resulted in a viable birth after the pregnancy was allowed to continue [6–10], uterine rupture in the third trimester and maternal death from intraoperative hemorrhage have also been reported, showing the risk of pregnancy continuation to be very high. Fortunately, our patient did not experience uterine rupture and had a viable birth. However, she did experience critical hemorrhage during cesarean section due to placenta percreta. When patients with CSP elect to continue a pregnancy, detailed informed consent concerning its risks must be obtained.

Making an accurate diagnosis in early pregnancy is critical for cases with a cesarean scar pregnancy progressing into the uterine cavity. Cesarean section is associated with a subsequent risk of cesarean scar pregnancy. Since the diagnosis is difficult except in early pregnancy, every woman with a previous cesarean section should be instructed to visit a medical facility soon after confirmation of pregnancy.
